# The impact of gender in the COVID-19 pandemic

**DOI:** 10.1192/j.eurpsy.2021.166

**Published:** 2021-08-13

**Authors:** S. Oertelt-Prigione

**Affiliations:** Department Of Primary And Community Care, University of Radbout, Nijmegen, Netherlands

**Keywords:** sex, COVID-19, Gender

## Abstract

Data worldwide is pointing towards an increased mortality of men a from COVID-19, while infection rates are equal or higher in women. Immunological differences might play a role in this as well as differences in risk factors and co-morbidities. In addition, differences in exposure, testing, case definitions and access to healthcare might play a role. Differences in symptoms have been reported, as well as potential differences in therapeutic choices. Also, the phenomenon of “long COVID” with all its psychophysical consequences appears to be more common in women. In addition to the consequences of the acute infection, COVID-19 is significantly impacting economies, social systems and political priorities. I will try to give a general overview of the current situation, starting from a medical standpoint and moving into the wider social consequences of this pandemic. I will highlight how the lack of attention to sex and gender can impact statistics, potential therapies and vaccines, livelihoods and the healthcare sector as a whole.

**Disclosure:**

No significant relationships.

